# A Quadruplex qPCR for Detection and Differentiation of Classic and Natural Recombinant Myxoma Virus Strains of Leporids

**DOI:** 10.3390/ijms222112052

**Published:** 2021-11-07

**Authors:** Fábio A. Abade dos Santos, Carina L. Carvalho, Francisco Parra, Kevin P. Dalton, Maria C. Peleteiro, Margarida D. Duarte

**Affiliations:** 1 Centro de Investigação Interdisciplinar em Sanidade Animal (CIISA), Faculdade de Medicina Veterinária, Universidade de Lisboa, Avenida da Universidade Técnica, 1300-477 Lisboa, Portugal; mcpelet@fmv.ulisboa.pt (M.C.P.); margarida.duarte@iniav.pt (M.D.D.); 2 Instituto Nacional de Investigação Agrária e Veterinária (INIAV, I.P.), Av. da República, Quinta do Marquês, 2780-157 Oeiras, Portugal; carina.carvalho@iniav.pt; 3 Instituto Universitario de Biotecnología de Asturias (IUBA), Departamento de Bioquímica y Biología Molecular, Universidad de Oviedo, 33003 Oviedo, Spain; fparra@uniovi.es (F.P.); daltonkevin@uniovi.es (K.P.D.)

**Keywords:** molecular diagnosis, multiplex, quadruplex qPCR, real-time PCR, *myxoma virus*, MYXV, natural recombinant MYXV, ha-MYXV, Iberian hare, European rabbit

## Abstract

A natural recombinant myxoma virus (referred to as ha-MYXV or MYXV-Tol08/18) emerged in the Iberian hare (*Lepus granatensis*) and the European rabbit (*Oryctolagus cuniculus*) in late 2018 and mid-2020, respectively. This new virus is genetically distinct from classic myxoma virus (MYXV) strains that caused myxomatosis in rabbits until then, by acquiring an additional 2.8 Kbp insert within the *m009L* gene that disrupted it into ORFs *m009L-a* and *m009L-b*. To distinguish ha-MYXV from classic MYXV strains, we developed a robust qPCR multiplex technique that combines the amplification of the m000.5L/R duplicated gene, conserved in all myxoma virus strains including ha-MYXV, with the amplification of two other genes targeted by the real-time PCR systems designed during this study, specific either for classic MYXV or ha-MYXV strains. The first system targets the boundaries between ORFs *m009L-a* and *m009L-b*, only contiguous in classic strains, while the second amplifies a fragment within gene *m060L*, only present in recombinant MYXV strains. All amplification reactions were validated and normalized by a fourth PCR system directed to a housekeeping gene (*18S rRNA*) conserved in eukaryotic organisms, including hares and rabbits. The multiplex PCR (mPCR) technique described here was optimized for *Taqman*^®^ and *Evagreen*^®^ systems allowing the detection of as few as nine copies of viral DNA in the sample with an efficiency > 93%. This real-time multiplex is the first fast method available for the differential diagnosis between classic and recombinant MYXV strains, also allowing the detection of co-infections. The system proves to be an essential and effective tool for monitoring the geographical spread of ha-MYXV in the hare and wild rabbit populations, supporting the management of both species in the field.

## 1. Introduction

Planet Earth has around 34 global diversity hotspots, featuring exceptional concentrations of endemic species, of uncountable and indisputable value [1,2]. For the past several decades, particularly after 1970, these areas have been experiencing an exceptional loss of habitats [3]. The decline or even disappearance of key species from these hotspots has been identified as the main threat, leading to catastrophic cascading effects on the affected ecosystems [4]. The western Mediterranean Basin hotspot holds a particularly high taxon diversity, at a global level, only overpassed in importance by the tropics [5]. In fact, in this area one can find the same plant richness (30,000 taxa) of all tropical Africa, which presents an area four times larger. In the western Mediterranean Basin hotspot 10.8 species/1000 km^2^ can be found, a much higher value than the homologous number in countries such as China, Zaire, India or Brazil [6]. The relevance of the wild rabbit (*Oryctolagus cuniculus*) for the maintenance of the Mediterranean scrubland of south-western Europe is such, that some ecologists named this ecosystem “the rabbit’s ecosystem” [7]. The wild rabbit and the Iberian hare (*Lepus granatensis*) are two of the most iconic and important species of the Iberian Peninsula, where they play a unique ecologic, cultural, and economic role.

Myxoma virus is a *Leporipoxvirus* from *Chordopoxvirinae* subfamily and *Poxviridae* family [8]. The myxoma virus (MYXV) possesses a large, linear double-stranded DNA genome with terminal inverted repeats (TIRs) and covalently closed hairpin loops at each end [9]. The Lausanne strain, the reference strain of classic Myxoma virus, encodes 158 unique open reading frames (ORFs), 12 being duplicated in the 11,577 bp-long TIRs [10,11].

In late 2018, a natural recombinant *myxoma virus* (hereinafter referred to as ha-MYXV, but also known as MYXV-Tol08/18) emerged in the Iberian hare (*Lepus granatensis*), affecting several populations in Spain [12,13,14] and Portugal [15] with an apparent mean mortality rate of 55.4% [14], and remarkable geographic dissemination, extending to all the Iberian peninsula in the first year of the emergence [14,16]. The ha-MYXV outbreaks raised additional concerns on the resilience limit of wild Iberian hare populations, due to their cumulative effect with the many insidious factors that have been responsible for the decline of both species in recent decades [17]. Despite the conservation status of the Iberian hare presently being of “Least Concern” by the International Union for Conservation of Nature (IUCN), many local populations are currently threatened as the result of a severe loss of habitat imposed by human activities, or by other infectious agents such as *Leporid gammaherpesvirus 5* (LeHV-5) that causes severe skin lesions with consequences on reproduction, or the cysticercosis, among others [18,19].

The ha-MYXV was initially detected only in Iberian hares suggesting a species-specificity for this virus. However, in mid-2020 it was also reported in diseased wild and domestic rabbits [20,21]. This finding questioned the effectiveness of current commercial vaccines against this new virus as well as the power of cross-immunity conferred by infection by the classic strains.

Furthermore, the susceptibility of the European rabbit to ha-MYXV brings additional worries towards the conservation of the species, attributed in 2019 the status “Endangered of Extinction” by the IUCN for the first time in history [22].

In the present context, the quick diagnosis of myxomatosis in wild leporids is of paramount importance for the successful management of the disease in the field, highly dependent on an early, prompt, and accurate diagnosis, given the rapid spread of the disease via direct and indirect contact between sick and healthy animals, the latter mediated by insects. For example, the laboratory confirmation of myxomatosis in hunting reserves, increases the managers’ awareness to intensify active prospection and removal of sick or dead animals from the field, thus reducing the sources of infection and contamination. In some cases, the control of the disease can be accelerated by capturing and vaccinating animals.

The multiplex molecular method (mPCR) here described constitutes an essential and effective tool for (i) the quick diagnosis of myxomatosis in rabbits and hares, (ii) the differentiation of classic MYXV strains from recombinant MYXV strains (ha-MYXV), and (iii) the detection of co-infections by both virus strains while showing the capacity/fitness to be adapted to *Taqman* or *Evagreen* systems, depending on the resources available in the laboratory and technicians’ preferences.

## 2. Results

### 2.1. Multiplex PCR Strategy

To optimize the diagnosis of myxomatosis, and differentiate classic MYXV from ha-MYXV strains in biological samples from European rabbit and Iberian hare, we developed a real-time quantitative multiplex PCR system (mPCR) by combining the qPCR system previously described by [23], which detects the *m000.5L/R* duplicated gene, with two other qPCR systems developed for this study. One of those systems targets the boundaries of the 2.8 Kbp insertion within gene *m009L* (that was disrupted in the ha-MYXV genome, abrogating amplification by this system), and the other (*m060L* system) targets a small region within gene *m060L*, solely present in ha-MYXV strains. The amplification and fluorescence results expected with the different infection situations were summarized in Table 1.

The Table 2 contains the genomic location of the targets and sequences of the primers and probes used in the mPCR.

The 2.8Kbp insert, located around 12,335 nt from the left end of the genome (in sequence KY548791), comprises a set of six genes, namely *m060L*, *m061L*, *m064L*, *m065L* and M066L, some of which are truncated. These genes are transcribed to the left, showing similarity with genes *m060R*, *m061R*, *m064R*, *m065R* and *m066R*, present downstream in the genome between position 57,321 and 61,760 (in sequence KY548791), which are transcribed to the right.

The duplicated *m000.5L/R* gene, located in the inverted terminal repeats (TIR) at the 5′ and 3′ ends of the Myxoma virus genome, is well conserved in all classic strains as well as in ha-MYXV, conferring high sensitivity to the method developed by Duarte et al., (2014) [23], which robustness and specificity were revalidated during this study. Interestingly, the duplicated *m000.5L/R* gene is absent in the high virulent MSW strain of Californian MYXV, which also shows a deletion of 845 bp in the 3′ end of the *m009L* gene (based on the Lu sequence) [24].

An internal reference targeting the *18S rRNA* housekeeping gene was used as a reliable gene [25] to control the nucleic acid extraction process and the efficiency of each amplification reaction. The *18S rRNA* is largely used as an internal control in diagnostic tests for the detection of human and animal RNA viruses [26]. The 18S rDNA codes for the small subunit of ribosomes and is a well-preserved gene among different species within the vertebrate phylum [27] being recently validated to be used in rabbit and hare’s tissues [28].

The probes’ fluorophores were selected to ensure that the fluorescence of the internal control (18S) probe would be lower than that of the other probes to reduce, as much as possible, the interference with the probes directed to viral DNA. Therefore, the CY5 fluorophore was chosen for labelling the 18S probe and the minimum concentrations of the probe and primers were determined, as long as a clear reading of the results was maintained. All the quenchers used were Black Hole Quenchers (BHQ™), to avoid fluorescent quenchers (e.g., TAMRA™).

### 2.2. Specificity of the Primers and Probes

The specificity of the three PCR systems (m000.5L/R, m009L and m060L) was first determined in silico, against the representative sequences available in the NCBI database, by using the BlastN analysis service from NCBI. Despite the evaluation of the specificity based only on the number and position of oligomers’ mismatches can be misleading [29], in silico validation of polymerase chain reaction primers and probes is a common procedure [30] to predict the functionality of the amplification reactions.

Therefore, to further confirm the specificity of these three systems, the nine oligomers were tested in silico (September, 2021) for potential annealing with the genomes of rabbit and hare pathogens with very relaxed temperature conditions (55 °C), showing all matching sites of primer binding, a length of 60 bp to 3000 bp for the amplicon and allowing mismatches in 5 nucleotides of the 3′ end), looking for all possible connection points using the software FASTPCR 6.7 (PrimerDigital, 2020). The pathogens investigated included *Rabbit fibroma virus* (NC_001266), *Goatpox virus* (NC_004003), *Murmansk poxvirus* (NC_03546), *Squirrel poxvirus* (NC_022563), *Yoka poxvirus* (NC_015960), *Murmansk poxvirus* (MF001304), *Teiidae poxvirus* (MT712273), *Cheloniid poxvirus 1* (MT799800), *Bibersteinia trehalosi* (NZ_CP006954), *Chlamydophila abortus* (CR848038), *Coxiella burnetii* (CP040059), *Cryptosporidium parvum* (CM000436), *Escherichia coli* (AE014075.1), *Encephalitozoon cuniculi* (LFTZ01000003), *Enterococcus faecalis* (CP045918), *Francisella tularensis* (CP025778), *Klebsiella pneumoniae* (FO203501), *Leptospira interrogans* (CP039256), *Mannheimia haemolytica* (CP006957), *Pasteurella multocida* (CP031552), *Salmonella enterica* (CP003278), *Serratia* sp. (CP025085), *Staphylococcus aureus* (AP017922), *Staphylococcus* epidermidis (CP043847) and *Toxoplasma gondii* (U87145). Despite viral RNA not being amplified by this multiplex PCR, a few RNA genomes were also evaluated given the possibility, in the future, of including these primers on a wider multiplex PCR System for RNA and DNA viruses. The three RNA viruses evaluated included *hare lagovirus* (KR230102.2 and MK138384), RHDV (MF421574) and EBHSV (MK440616).

The putative results of the quadruplex system deduced from the BLAST analysis are shown in Table S1. From a total of 85 genomes, including all the representative strains of myxoma virus available in the NCBI database since 1949 (until September 2021), only one strain was not detected by this system, namely the California/San Francisco MSW strain, isolated in 1950 (KF148065). So far, the recent ha-MYXV strains are unique being detected by the M060L system.

The low variability of the *m000.5L/R* gene, and its duplication at the opposite ends of the genome, makes it an excellent candidate to assure that, if present, all MYXV strains are detected. On the contrary, the *m009L* gene is prone to mutations, deletions and additions, making it ideal to detect the current European MYXV strains and to evaluate genetic variability and virus evolution.

None of the pathogens’ sequences and samples listed in this and 4.4.1 subsections, analysed in silico and in vitro, respectively, were detected by any of the three methods. Likewise, in silico analysis of the heterologous vaccines (Shope Fibroma Virus vaccines, namely Mixohipra FSA, Mixovacina and Lyomyxovax) showed that none of these vaccines was detected by any of the three systems. In the case of homologous vaccines (Myxo-RHD Plus, Dervaximyxo, Mixohipra-H, POX-LAP and Dercunimix SG33), none was detected by the *m060L* system as expected, all vaccines were detected by the *m000.5L/R* system, and only Dervaximyxo and Dercunimix vaccines were detected by the *m009L* system (Table S1).

Most homologous vaccines underwent genetic modifications in the *m009L* gene during cell culture passages, or as a result of natural changes or genetic manipulation of the virus strains, to attenuate the virus. In fact, during this study we sequenced a few commercial vaccines, confirming this variability (not shown for confidentiality reasons). Regarding the European classic field strains, except for one strain from Germany (1985, KP723387), one strain from Spain (1995, EU552530) and two strains from England (2009, KY548812 and 2011, KY548813), the target regions of the *m009L* primers and probe are fully conserved in all the classic strains which sequences are available in the NCBI (Table S1). On the contrary, in the ha-MYXV strains the *m009L* region is interrupted by the insertion of a 2.8 kbp additional sequence, separating the two target sequences of the *m009L* primers by around 3.0 kb, and interrupting the probe-target sequence, therefore hampering annealing. Consequently, the *m009L* method does not detect these strains, leading to negative results. The positivity in the *m000.5L/R* system excludes false-negative results for myxomatosis.

### 2.3. Limit of Detection and Sensitivity of the Uniplex Systems

**Figure 1:**
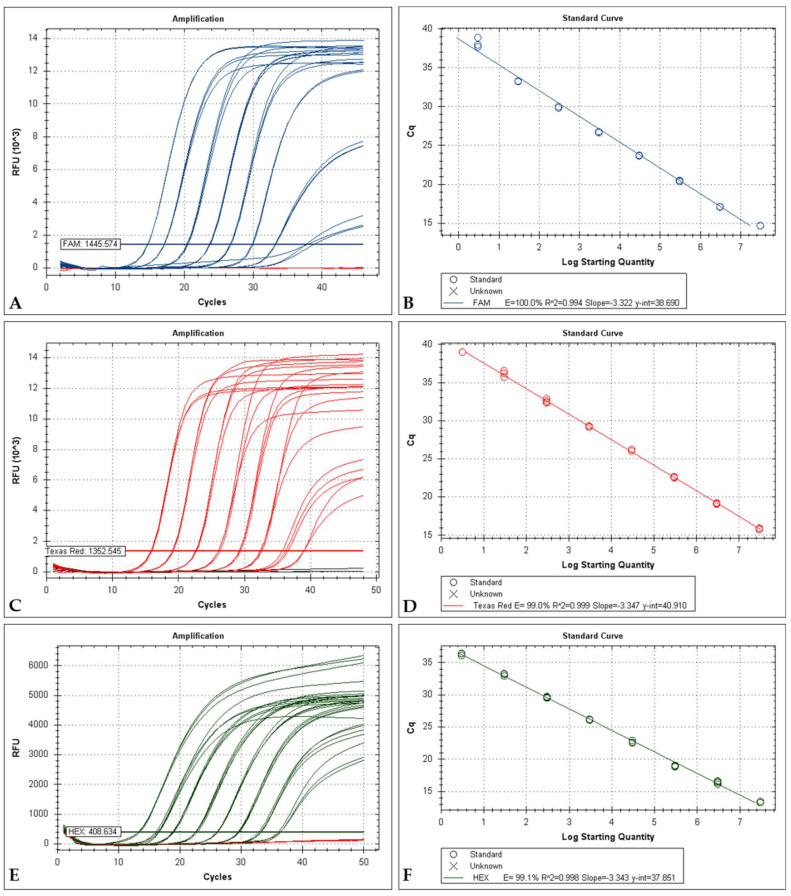
(**A**) Serial dilution from 3.0 × 10^7^ to 3.0 × 10^0^ copies of *m000.5L/R* per reaction. Red lines correspond to negative controls (3.0 × 10^−1^ copies). (**B**) Standard curve for the *m000.5L/R* qPCR, the log starting quantity ranged from 3.0 × 10^7^ to 3.0 × 10^0^ copies in the final reaction. (**C**) Serial dilution from 3.0 × 10^7^ to 3.0 × 10^0^ copies of *m060L* per reaction. Black lines correspond to negative controls (3.0 × 10^−1^ copies) (**D**) Standard curve for the *m060L* qPCR, the log starting quantity ranged from 3.0 × 10^7^ to 3.0 × 10^0^ copies in the final reaction. (**E**) Serial dilution from 3.0 × 10^7^ to 3.0 × 10^0^ copies of *m009L* per reaction. Red lines correspond to negative controls (3.010^−1^ copies). (**F**) Standard curve for the *m009L* qPCR, the log starting quantity ranged from 3.0 × 10^7^ to 3.0 × 10^0^ copies in the final reaction.

The minimum copy number of MYXV DNA detected by the *m000.5L/R* uniplex PCR was 3 copies, following the value previously described (2.6 copies) by Duarte et al. [23]. Both the *m060L* and *m009L* systems demonstrated to be able of detecting as few as 3 copies of viral DNA. In the uniplex modality, the amplification efficiency for the *m060L*, *m009L* and *18S rRNA* targets was above 98% (Figure 1).

Typical standard curve amplification plots and linear regression analyses were found for the three singleplex (Figure 1). Excellent linearity was observed over 8 orders of magnitude, from 3 × 10^7^ to 3 × 10^0^ copies. The regression analyses for these intervals yielded R^2^ (correlation coefficients) of 0.99. The slopes around 3.3 revealed high qPCR efficiency (Figure 1B,D,E).

### 2.4. Determination of the mPCR Optimal Conditions and 3D Plot Analysis for the Quadruplex System

**Figure 2:**
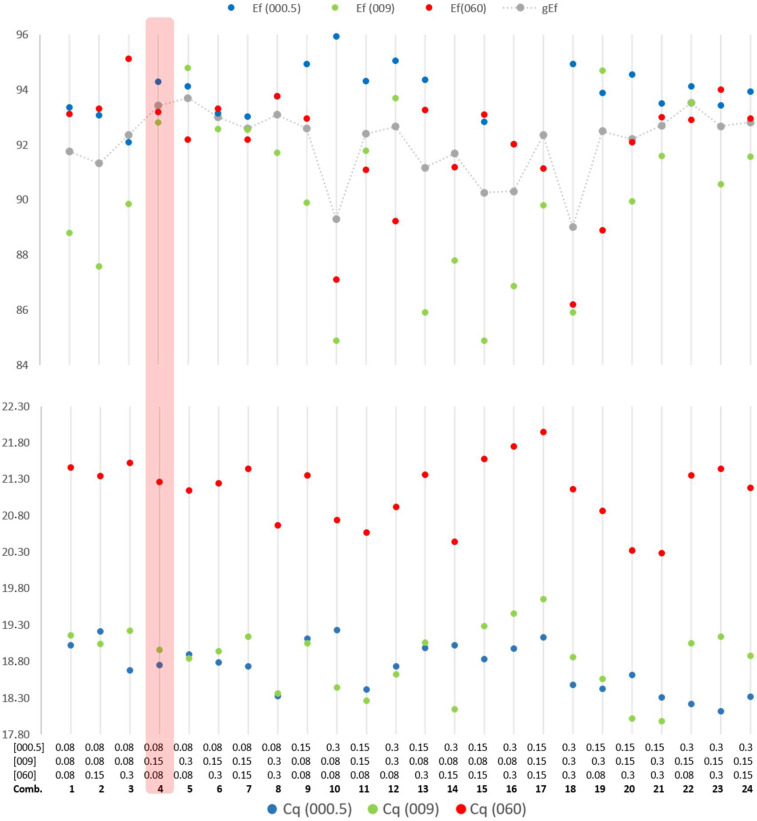
Graphical presentation of mPCR performance using different primers concentrations ([m000.5], [m009], [m060]) The grey dots are joined together (therefore not representing a trend line) to illustrate the variation in average efficiency.

The initial optimization of the primers and probe concentrations in the mPCR modality revealed an expected cross-effect between the different systems concerning the concentrations of the primers. Figure 2 illustrates the optimization of primer concentration of each system to obtain balance in the amplification efficiency of the three systems and Cycle quantification.

All combinations of variable primers and probes concentrations that generated amplification efficiencies below 90% for the three systems, or at least for one, were excluded. This was the case for combinations number 1, 2, 3, 10, 13, 14, 15, 16, 17, 18 and 20, illustrated in Figure 2. As shown in Figure 2, combination 4 was chosen based on the good efficiency, adequate Cq values, and sigmoidal curve shapes (not visible in the figure). This combination was then extensively tested and optimized for small variations in the concentrations of primers and probes (results not shown) to balance the fluorescence between the four systems (avoiding fluorescence saturation).

The selected conditions based on the 3D Plot analysis were annealing temperature of 60 °C, the Mg^2+^ concentration recommend by the kit manufacture (2.5 mM) and the dNTP concentration kept to the minimum (0.2 mM).

### 2.5. Primer and Probes—Recommended Concentrations and mPCR Protocol

The final concentrations for the primers and probes and the optimized protocols for the mPCR are shown in Table 3 and Table 4, respectively. However, these conditions should be optimized for other enzymes and devices.

### 2.6. Detection Limit and Sensitivity of the Optimized mPCR

**Figure 3:**
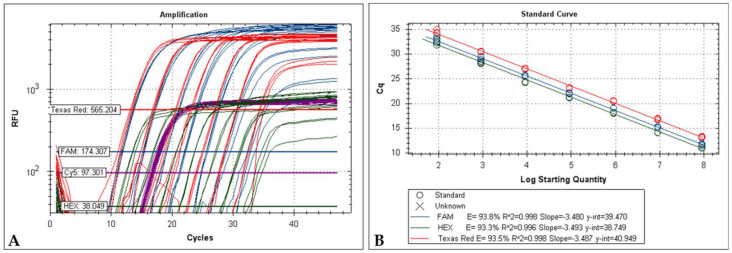
(**A**) Serial dilution from 9.0 × 10^7^ to 9.0 × 10^1^ copies of each plasmid (pM000.5L/R, pM009L and pM060L) and a constant 3 × 10^6^ copies of p18S per reaction. Red curves (TexRed) correspond to *m060L* system, blue curves (FAM) corresponding to *m000.5L/R* system, green curves (HEX) corresponding to *m009L* system and purple curves (CY5) correspond to 18S system. (**B**) Standard curve for the quadruplex qPCR. The reaction copies ranged from 9.0 × 10^7^ to 9.0 × 10^1^ copies in the final reaction.

The mPCR system was able to detect as few as 9 molecules of DNA per 50 μL of PCR reaction of *m000.5L/R*, *m060L* and *m009L* genes. The ten-fold serial dilutions standard curves generated by the quadruplex system corresponding to 9.0 × 10^7^ to 9.0 × 10^1^ copies per reaction are shown in Figure 3, resulting from an average of 3 standard curves for each target. The dilutions of 9.0 × 10^0^ were excluded from the standard curve analysis because of the scale, curve shape and high coefficient of variation.

### 2.7. Intra and Inter-Assay Variation

To evaluate intra-assay variability or repeatability of the qPCR quadruplex, the coefficient of variation (CV%) was calculated using the Cq values from the quadruplicates of each dilution. The maximum CV determined was 3.65%, close to the ideal maximum value of 3.5%. To access plate-to-plate consistency, the inter-assay variation or reproducibility, the %CV was calculated using the Cq values from duplicates in two different runs. The inter-assay variability demonstrated the good reproducibility performance of the multiplex with Cq values generating CVs under 3.52%. The Cq mean standard deviation and CV% values obtained with the mPCR are summarized in Table 5.

### 2.8. Eva Green-Based Multiplex Real-Time PCR (EG-mPCR)

**Figure 4:**
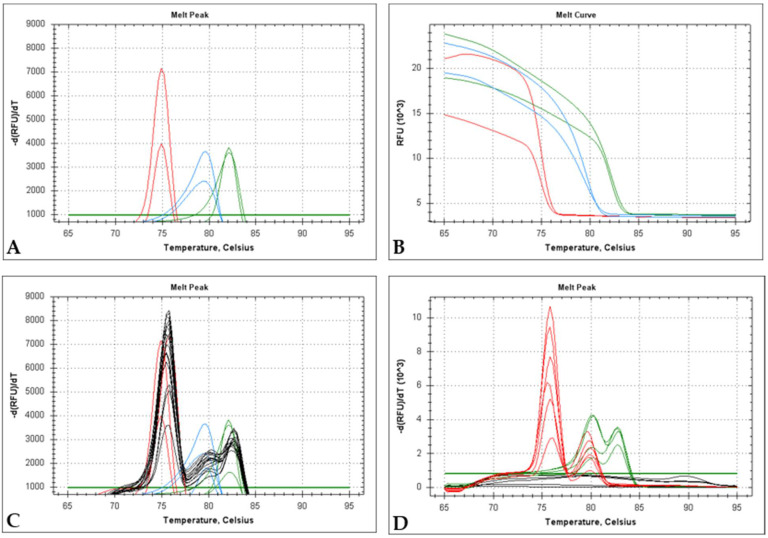
(**A**) Melting peaks of EvaGreen-PCR in singleplex reactions. Red lines, blue lines and green lines correspond to *m060L*, *m000.5L/R* and *m009L* systems, respectively. (**B**) Dissociation curves from (**A**). (**C**) Melting peaks analysis for the three systems described in (**A**,**B**), superimposed by the respective triplex analysis (three plasmids in the reaction, black lines), with the correspondent three peaks. (**D**) Melting peaks analysis for field samples using the EvaGreen-mPCR, where red lines represent samples positive to ha-MYXV and green lines samples positive for classic MYXV strains.

The mPCR was adapted to EvaGreen to allow its use without the need of purchasing expensive Taqman probes. In singleplex amplification, three distinct peaks (Figure 4A) were observed corresponding to good dissociation curves (Figure 4B). Figure 4A shows the Evagreen amplification peaks in the Evagreen systems of two out of the eight dilutions tested of plasmids pM060L, pM000.5 L/R and pM009L.

Triplex analysis using eight tenfold dilutions of the same plasmids generated three distinct peaks (Figure 4C, black lines), homologous to those observed in the singleplex reactions (Figure 4A,C).

When field samples ha-MYXV-positive were tested, two peaks were observed corresponding to the *m060L* (left red peak) and *m000.5L/R* (right red peak) genes specific amplification. Two peaks were also detected with classic MYXV-positive samples, corresponding to the amplification of the *m000.5 L/R* (left green peak) and *m009L* (right green peak) genes. The melting temperatures of each one of the three amplicons proved to be highly stable and differentiated, regardless of the modality of the qPCR used (Table 6).

The The melting temperature peak for each system was determined by the mean ±SD of four duplicates replicated in two different runs.

### 2.9. Field Sample Analysis

**Figure 5:**
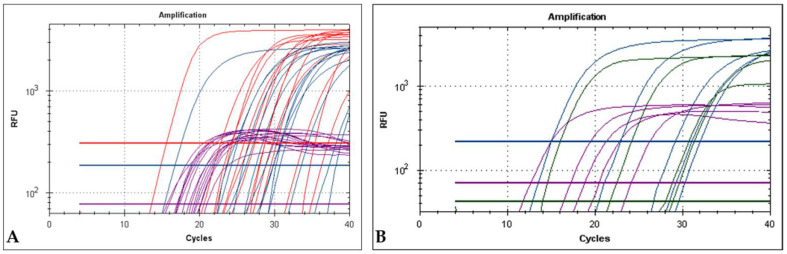
(**A**) Results in field samples positive to ha-MYXV (red sigmoid curves: *m060L*, Texas Red channel; blue sigmoid curves: *m000.5L/R*, FAM channel; green flat lines: m009L, HEX channel, not visible due to scale). (**B**) Results with field samples positive for classic MYXV (blue curves: *m000.5L/R*, FAM channel; green curves: *m009L*, HEX channel, red flat lines: *m060L*, Texas Red channel, not visible due to scale). All samples were positive for 18S rDNA amplification (CY5, purple curves).

To validate the developed mPCR system for routine diagnosis, the multiplex system protocol with Taqman probes was used to test field samples from National Institute for Agricultural and Veterinary Research—INIAV-biobank, previously classified as positive or negative for MYXV and ha-MYXV using the clinical and necropsy data, a qPCR recommended by the OIE Terrestrial Manual [31] and a conventional PCR previously published [12]. A sample is considered ha-MYXV-positive (Figure 5A) in the mPCR when the qPCRs targeting the *m000.5L/R*, *m060L* and *18S rRNA* genes generate fluorescence signals with Cq values below 38.0, and the *m009L* system does not amplify DNA from the sample (Table 2). On the other hand, a sample is considered classic MYXV-positive (Figure 5B) when the qPCR targeting the *m000.5L/R*, *m009L* and *18S rRNA* genes generate fluorescence signals with Cq values below 38.0, and the M060L system does not amplify DNA from the sample been tested (Table 2). Classic MYXV and/or ha-MYXV positive samples generate exponential fluorescence in channels CY5 (18S rDNA amplification, Cq values < 38.0) and FAM (*m000.5 L/R* amplification). Using the mPCR system developed, we were able to confirm the results previously obtained regarding 59 out of the 60 samples tested (98.33%) (Table 7). For one classic MYXV-positive sample, tested in duplicate, no amplification was obtained with the m009L system in the quadruplex PCR, probably due to the lower efficiency of the system compared to the singleplex. Nevertheless, this sample showed a positive result in the *m000.5 L/R* system (Cq 35.2, considered as very low viral load), confirming the presence of MYXV-DNA in the sample. When in uniplex, amplification of this sample with *m009L* was successful (Cq 37.1). None of the field samples analysed so far by our team was simultaneously positive for both viruses, indicating that co-infection by MYXV and ha-MYXV is not a common event.

The lower fluorescence of the 18S PCR system compared with the fluorescence reached with the *m060L*, *m000.5 L/R*, and *m009L* qPCRs (Figure 5) was imposed by limiting the primers and probe concentrations. Despite this, the CY5 low fluorescence does not compromise the validity of the extraction and PCR, and minimizes the interference between channels and fluorescence saturation.

## 3. Discussions

Wild leporids are an iconic species of the Iberian Peninsula, where they have tremendous ecological, cultural, and economic importance. Myxomatosis has been a severe menace for the wild rabbit since it emerged on the Iberia Peninsula in 1980. In 2018, the disease emerged for the first time in the Iberian hare [15,32] causing high mortality in the wild populations and aggravating the decrease of this species in both Portugal and Spain [19]. In Portugal, myxomatosis accounted for 27.8% of the mortality of wild rabbits verified in the field between mid-2017 and late 2020 (data from the Action Plan for the control of RHDV2 in wild rabbits, dispatch 4757/17 of May 31) and for 88% of the mortality of Iberian hares (after the emergence of ha-MYXV in October 2018), accelerating the reduction of the wild populations of both species that have been observed in recent decades, also, as a result of land-use changes.

The impact of myxomatosis in the wild leporids populations makes the existence of a rapid, less laborious and low-cost tool for laboratory diagnosis of the disease and virus discrimination/characterization in myxomatosis outbreaks in these species imperative. Here we describe the first real-time PCR method to detect ha-MYXV strains that simultaneously enables investigating the presence of classic MYXV strains and putative co-infections. The three PCR systems that are integrated into the mPCR are validated by the amplification of internal control (*18S rRNA* gene), previously demonstrated to be a strong and stable housekeeping gene (Abade dos Santos et al., 2021). The sensitivity and specificity of these PCR systems were 100% when samples previously classified as positive or negative were screened by the *m000.5L/R*-based system [23] and further classified as recombinant/classic MYXV by the system developed by [12].

When the four systems were used together in the quadruplex, the mPCR proved to be robust, highly sensitive and highly specific. The technique has the advantage of being applied using two different PCR formats, according to the laboratory preferences, namely by hydrolysis of a Taqman probe or green fluorescent nucleic acid dying (EvaGreen). Theoretically, the cycle quantification values in the multiplex (shown in Figure 3 and Table 5) and uniplex assays (shown in Figure 1) should be superimposable. However, during the in vitro assays, the efficiency interference and the fluorescence crossover between probes deviate from these values, despite still being relatively close.

The *m060L* system is directed to a gene of the ha-MYXV 2.8 kbp insert that is homologous to *m060R* gene, located further downstream in both classic MYXV and ha-MYXV genomes. However, as the primers and probes target sequences within the *m060R* gene, present a high number of mismatches (>9) in classic MYXV strains (Lausanne strain, KY548791) no amplification occurs under the conditions used. Even under very permissive conditions (such as an annealing temperature of 56 °C, and 40 cycles) neither exponential amplification was detected in the real-time PCR nor amplicon was synthesized using the primers in a conventional PCR format. We demonstrated the absence of amplification of the *m060R* gene with the oligomers of the *m060L* system in 20 samples from classic MYXV infected rabbits. The mPCR method has two levels of internal validation, through the *m000.5L/R* and *18S rRNA* systems. Amplification of the 18S rDNA target [28] validates the quality of tissue, the extraction and amplification efficiency, while amplification of the duplicated *m000.5L/R* target, allows for the presumptive initial diagnosis of the infection. Furthermore, the amplification of target *m000.5L/R* allows the exclusion of potential recombination in genes *m009L* and *m060L* that could lead to false-negative results. m009L negative results could also occur due to mutations in this gene, as verified in silico for some strains, namely Australian strains and vaccine strains. Only the Dervaximyxo and Dercunimix were detected by in silico analysis using the *m009L* system (Table S1). Despite the gene *m000.5L/R* being duplicated, the copy number must be similar to the other target genes, meaning that the Cq value should be similar (Cq value of *m000.5L/R* similar to Cq value of *m009L* in animals infected with classic MYXV and Cq value of *m000.5L/R* similar to Cq of *m060L* in animals infected with classic MYXV), apart from the difference in their efficiencies and threshold line (Table 5).

A Cq value in the 18S rDNA channel ≥ 30.0 could mean problems with the original sample quality (e.g., degradation or over dilution), in the extraction or during the PCR (e.g., inhibitors). Simultaneous amplification of *m000.5L/R*, *18S rRNA*, *m009L* and *m060L* could mean co-infection with classic and recombinant MYXV.

Although no leporids were found co-infected by classic and recombinant strains of MYXV, this situation was simulated in three different ways: (i) with samples containing the four plasmids (pM000.5, pM060, pM009 and p18S), (ii) with nucleic acids extracted from a mixture of eyelids from rabbits infected with the two viruses and (iii) with a mixture of DNA obtained separately from the two strains. In all three situations, simultaneous amplification of MYXV and ha-MYXV DNA took place either using the Taqman or the Evagreen system.

*m009L* is a frequently mutated gene as shown by the BLAST analysis (Table S1), with some geographic related variations, such as single deletions or substitutions [33] in the Australian strains. In some strains, these genetic mutations prevent the amplification by the *m009L* system (Table S1). The *m009L* gene is also a target of larger deletions, for example in strain 6918 [11], despite not affecting the m009L detection. Since *m009L* was the target of the main genetic change found in the ha-MYXV strains, it was chosen for the mPCR, functioning as a monitor of this specific genetic region. In fact, of all the representative sequences currently available in the NCBI database, all classic MYXV Europe strains (except the four strains referred to in Section 2.2) would be detected in the m009L system (Table S1). On the other hand, using the *m000.5L/R* system in parallel, the detection of the virus is guaranteed even in the absence of virus detection by the *m009L* or *m060L* systems due to mutations.

The systemic distribution of the MYXV during infection allows the diagnosis to be performed using different organs, although with variable Cq values (e.g., lower in the skin with lesions, and higher in spleen and liver). Additionally, given the need to perform diagnosis in wild animals, often undergoing some degree of degradation and autolysis, we recommend that samples with Cq values over 38.0 but with a sigmoid shape, be further investigated by repeating the analysis using the duplex system (18S and target amplicon) before being considered negative.

To our best knowledge, this is the first real-time PCR method for ha-MYXV diagnosis in both rabbits and hares and proved to be a robust technique for differentiation between classic and natural recombinant strains using two alternative systems, according to preference.

## 4. Material and Methods

### 4.1. Biologic Samples and DNA Extraction

A total of 60 samples from the biobank of the National Reference Laboratory for Animal Diseases (INIAV I.P.), collected between 2017 and 2021 from wild leporids all over the Portuguese national territory were used in this study. The samples had been previously tested for myxomatosis by molecular methods and submitted to the anatomopathological examination and in vitro virus propagation [16,23,34].

For nucleic acid extraction, fresh or frozen samples of tissues from of the eyelids and liver/spleen from ha-MYXV or classic MYXV positive leporids were homogenized at 20% (*w*/*v*) with phosphate-buffered saline (PBS) and clarified at 3000× *g* for 5 min. Total DNA and RNA were extracted from 200 μL of the clarified supernatants using the MagAttract 96 cador Pathogen Kit (Qiagen, Hilden, Germany) in a BioSprint 96 nucleic acid extractor (Qiagen, Hilden, Germany), according to the manufacturer’s protocol. Nucleic acids were preserved at −20 °C until use.

### 4.2. Primers and Probes Design

During this study, a Taqman probe system was designed to specifically detect ha-MYXV strains based on the amplification of a 178 bp-long genomic region corresponding to part of gene *m060L*, only present in the 2.8 Kbp insert.

For the detection of classic MYXV strains, a Taqman probe system was designed based on the amplification of an internal region of gene *m009L*. This 146 bp-long region is only present in the genome of classic myxoma virus strains since it encompasses the zone disrupted by the 2.8 Kbp insertion in ha-MYXV strains

Primers and probes were designed with the PCR Primer Design software (Eurofins Genomics, Ebersberg, Germany) and manually optimized, based on the alignment of six recombinant MYXV strains’ sequences obtained from Iberian hares (MK340973 and MK836424), domestic rabbits (MT920563 and MT920564) and wild rabbits (MT940239 and MT940240). The nucleotide sequences of the nine oligomers used are described in Table 2.

The four systems (*m000.5L/R*, *m009L*, *m060L* and *18S rRNA*) were manually optimized to allow the use of the same annealing temperature in the multiplex assay and to generate amplicons with differentiable melting temperatures necessary for melting curve analysis.

### 4.3. Cloning and Sequencing of the qPCR Amplicons

Amplicons were obtained by conventional PCR with the four sets of primers mentioned above, using DNA extracted from a ha-MYXV positive hare (for *m060L* amplification), an MYXV-positive rabbit (for *m000.5L/R* and *m009L* amplification) and a healthy wild rabbit (for *18S rRNA* amplification). Amplifications were carried out using the FastStart TaqMan Probe Master Kit (Roche, Roche Diagnostics GmbH, Manheim, Germany), in a 25 μL final reaction volume using 12.5 μL of 2× buffer, 0.5 μL of forward primer (0.2 µM final concentration), 0.5 μL of reverse primer (0.2 µM final concentration), 5 μL of DNA template and 6.5 μL of RNase/DNase free water. Denaturation and extension conditions were carried out according to the instructions in the PCR kit, using an annealing temperature of 60 °C. The qPCR reactions were conducted in a Bio-Rad’s CFX96 Dx Real-Time PCR equipment.

The size of the amplicons was confirmed by agarose gel electrophoresis, and the reaction products were directly cloned into the pCR2.1 TA vector (Invitrogen Corporation, San Diego, CA, USA) accordingly with the manufacture’s recommendations. Five recombinant plasmids containing the inserts (pM000.5 L/R, pM060L, pM009L, p18S(hare), p18S(rabbit)) were purified from *Escherichia coli* recombinants grown in Luria Broth medium, at 37 °C with 220 rotations per minute, using the NZYMiniprep Kit (NZYTech, Lisbon, Portugal). Both strands of the inserts were cycle sequenced using the M13F and M13R primers and a 3130 Genetic Analyser (Applied Biosystems, Foster City, CA, USA). Sequences were assembled and edited using the BioEdit 7.2 software (Bioedit, Manchester, UK). 

### 4.4. Detection Limit, Sensibility, Specificity, Repeatability and Reproducibility

#### 4.4.1. Specificity: In Vitro Analysis

Each one of the three qPCR was optimised individually. While the m000.5L/R system efficiency, sensitivity and specificity were previously demonstrated (Duarte et al., 2014), the specificity of the primers and probes for the *m060L* and *m009L* qPCR systems, were tested in vitro against total nucleic acids extracted from animal tissues infected with viruses including an avian poxvirus (*Fowlpox virus*) and leporid virus (*rabbit haemorrhagic disease virus*, *Leporid gammaherpesvirus 5*). Healthy rabbit and hare tissues, DNA from isolated from bacteria (*E. coli*, *P. multocida*, and *S. aureus*), and parasites (*Cysticercus pisiformis*, *Eimeria stiedae*, *Eimeria* spp. and *Passalurus ambiguous*), available at the National Reference Laboratory for Animal Health (INIAV, I.P, Oeiras, Portugal) were also tested. In all qPCR reactions performed, 20 ng of DNA template were used. Amplifications were performed using duplex systems which included the 18S internal control and one specific target at a time. A relaxed annealing temperature of 55 °C was used to decrease the specificity.

Additionally, all the available myxomatosis (with homologous and heterologous virus) vaccines were tested by in silico and/or in vitro analysis (Table S1). The conditions for in silico analysis were described above (Section 2.2).

#### 4.4.2. Detection Limit, Sensibility, Repeatability and Reproducibility

The DNA concentration of the purified recombinant plasmids was determined by A260 measurement (Qubit 4 Fluorimeter by Invitrogen) and the copy number calculated based on the plasmids molecular weight, where 3929 bp corresponds to the plasmid plus the insert (amplicon of interest).

After plasmid purification, the DNA concentration and purity of each plasmid (for each system) was distinct from the others. From each purified DNA, an initial dilution corresponding to 6 × 10^6^ copies/μL (corresponding to 3.0 × 10^7^ copies of DNA template in 5 μL, the volume added to the PCR reactions) was prepared and from these 10-fold serial dilutions were made. All the dilutions were performed using Nuclease-Free Water, for Molecular Biology (Sigma-Alrich, St. Louis, Missouri, USA). For each system, the standard curves were constructed using three replicates per dilution by plotting the cycle threshold (Cq) values against the logarithm of the DNA copy number. The absolute copy number of plasmid concerning the starting amount of DNA was calculated accordingly to the formula: Number of copies detected = amount of DNA (g) × 6.022 × 10^23^/fragment (bp) × 650.

The four singleplex PCRs were performed with the Multiplex PCR NZYTaq 2× Colourless Master Mix (NZYTech, Lisbon, Portugal) in a final volume of 25 μL, containing 12.5 μL of 2× buffer, 0.5 μL of forward primer (0.2 µM final concentration), 0.5 μL of reverse primer (0.2 µM final concentration), 0.5 μL of probe (0.2 µM final concentration), 5 μL of DNA template and 6.5 μL of RNase/DNase free water.

### 4.5. Optimization of the Multiplex Real-Time PCR Assay (mPCR)

After optimization of the four singleplex qPCR (*m000.5L/R*, *m060L*, *m009L* and *18S rRNA*), the final concentration of each of the eight primers and four probes combined was optimized in the mPCR to avoid cross-inhibition or interferences between the different fluorescence signals.

While maintaining the 18S primers and probe concentrations to a minimum (Table 3), their effect in the efficiency of amplification (comparing assays with or without the 18S system), average efficiency and Cycle threshold for the other three targets were evaluated by varying the concentrations of the primers and probes (Figure 2). The efficiency was estimated using three replicates (the mean value is shown the Figure 2) for three different concentrations of each target (9 × 10^5^, 9 × 10^4^ and 9 × 10^3^ copies per reaction), using the respective recombinant plasmids. The p18S plasmid was kept stable at 3 × 10^6^ copies per reaction. The Cq values shown in Figure 2 correspond to three replicates (average value presented) using 9 × 10^5^ copies of each plasmid (pM060, pM009, pM000.5) and 3 × 10^6^ copies of p18S.

The response surface method (RSM) was used to evaluate the effects of interactions between variables (primers, probe and Mg^2+^ concentration). For this analysis, a fixed number of copies (3 × 10^5^) of the recombinant plasmid was used. The conditions testes were dNTP concentration (0.2, 0.3, 0.4 mM), MgCl_2_ concentration (2.5, 3, 4, 5 and 6 mM), and annealing temperature (52, 52.7, 54, 55.9, 58.4, 60.3, 61.4, 62 °C). The optimal values were selected to balance the different intrinsic fluorescence of the fluorophores and the efficiencies of the different systems so that a balanced amplification and near fluorescence of the various targets occurs.

The final concentrations were still submitted to final optimizations to minimize the fluorescence differences between the probes and the different efficiency of the systems, in which the denaturation, annealing and extension times of the Taqman and Evagreen systems were also evaluated. All mPCR reactions were prepared in a final volume of 50 µL. The final conditions are shown in Table 3 and Table 4.

### 4.6. Field Samples Analysis

The sensitivity and specificity of the method were evaluated with 60 field samples obtained between 2017 and 2021 from the INIAV biobank, comprising 10 hare and 9 rabbit samples that had tested negative to MYXV and ha-MYXV, 17 rabbit samples that tested positive for MYXV, and 20 hare and 4 rabbit samples that tested positive to ha-MYXV. All samples were analysed in duplicate. The initial diagnosis was carried out by real-time directed to the *m000.5L/R* gene, a method recommended by the OIE [23] to detect MYXV, and by a conventional PCR [12], followed by sequencing to confirm the presence of recombinant strains containing the disruptive insertion within the *m009L* gene.

### 4.7. EvaGreen-Based Multiplex Real-Time PCR (EG-mPCR)

The Taqman multiplex system was also optimized for the SsoFast™ EvaGreen^®^ Supermix amplification kit (Bio-Rad, Lisbon, Portugal). The conditions were the following for a final 20 μL reaction: 10 μL of SsoFast EvaGreen supermix, 0.5 μL of each forward primer (0.08 to 0.12 μM, Table 3), 0.5 μL of reverse primer (0.08 to 0.12 μM, Table 3), 5 μL of DNA template and 4 μL of RNase/DNase free water.

Amplifications were run on a CFX96 real-time system associated with the C1000 thermal cycle (BIORAD), under the following conditions: an initial denaturation step at 98 °C for 2 min followed by 39 cycles of denaturation at 98 °C for 5 s, and annealing and extension at 60 °C for 15 s. Finally, the melting curve was analysed at a range of temperatures of 65–95 °C (with 0.3 °C increments), 5 s/step. The melting temperature of each amplicon (*m000.5L/R*, *m009L* and *m060L*) was initially determined by uniplex and then validated to the multiplex.

## Figures and Tables

**Table 1:** Expected results for the different targets in the mPCR. * the *m009L* system may lack the detection of some classical MYXV strains (see Table S1).

	Amplification and Fluorescence Detection			
	*m000.5L/R* qPCR	*m009L* qPCR	*m060L* qPCR	18S rDNA qPCR
MYXV	yes	yes/no *	no	yes
ha-MYXV	yes	no	yes	yes
Non-infected	no	no	no	yes
MYXV and ha-MYXV (Coinfection)	yes	yes/no *	yes	yes

**Table 2:** Genomic location of the targets and sequences of the primers and probes used in the mPCR. Homology against two standard sequences (Lausanne strain (KY548791), and Tol08-18 strain (MK340973)) is marked. Underlined nucleotides represent mismatches.

				Location and Conservation of the Primers and Probes’ Target Sequences in the Classic MYXV (Lausanne) and ha-MYXV (Tol08-18) Strains				
qPCR System	Oligomer (Fluorophore-Quencher)	Nucleotide Sequence (5′-3′)	Position in Sequence KY548791 (Lausanne Strain)	Homologous Sequence in KY548791 (Percentage of Similarity with Primer/Probe; Gene Targeted by the PCR System)		Position in Sequence MK340973 (Tol08-18 Strain)	Homologous Sequence in MK340973 (Percentage of Similarity with Primer/Probe, Gene Targeted by the PCR System)	
*m000.5L/R* (duplicated) [23]	Forward Primer	CGACGTAGATTTATCGTATACC	558 to 537 and 161,220 to 161,241	CGACGTAGATTTATCGTATACC (100%; *m000.5L/R* gene)	125 bp	564 to 543 and 163,997 to 164,018	CGACGTAGATTTATCGTATACC (100%; *m000.5L/R* gene)	125 bp
	Reverse Primer	GTCTGTCTATGTATTCTATCTCC	434 to 456 and 161344 to 161322	GTCTGTCTATGTATTCTATCTCC (100%; *m000.5L/R* gene)		440 to 462 and 164,121 to 164,099	GTCTGTCTATGTATTCTATCTCC (100%; *m000.5L/R* gene)	
	Probe (Fam/BHQ1)	TCGGTCTATCCTCGGGCAGACATAGA	483 to 508 and 161,295 to 161,270	TCGGTCTATCCTCGGGCAGACATAGA (100%; *m000.5L/R* gene)		489 to 514 and 164,072 to 164,047	TCGGTCTATCCTCGGGCAGACATAGA (100%; *m000.5L/R* gene)	
*m009L*	Forward Primer	TCCATTTACGATACACGCCGACGC	12,171 a 12,194	TCCATTTACGATACACGCCGACGC (100%; *m009L* gene)	146 bp	12,147 to 12,170	TCCATTTACGATACACGCCGACGC (100%; *m009L-a* gene)	No amplicon ^#^
	Reverse Primer	ACAACGTTCTATACTGTTTAGGGGGTACG	12,316 to 12,288	ACAACGTTCTATACTGTTTAGGGGGTACG (100%; *m009L* gene)		15,154 to 15,127	ACAACGTTCTATACTGTTTAGGGG-TACG (97%, 1 deletion; intergenic region *m009L-a* and *m009L-b*)	
	Probe (TexasRed/BHQ2)	TACGATCTACTGACGAACGAATACAGTTTAATGCC	12,254 to 12,220	TACGATCTACTGACGAACGAATACAGTTTAATGCC (100%; *m009L* gene)		15,093 to 15,059	TACGATCTACTGACGAACAATGGATCACGGAAAGT (57%; intergenic region *m009L-a* and *m009L-b*)	
*m060L*	Forward Primer	GATTCTTTAATCTGGTTGAGGCAACTA	57,669 to 57,643	TCTTTAATCTAGTCGTTGCGAGAACAA (48%; *m060R* gene)	No amplicon	14,723 to 14,749	GATTCTTTAATCTGGTTGAGGCAACTA (100%; *m060L* gene)	178 bp (*m060L*) and no amplicon (*m060R*)
						60,497 to 60,471	GTTTCTTTAATCTAGTCGTTGCGAGAA (70%; *m060R* gene)	
	Reverse Primer	GGATATTATTACGCTCCATTATCGGAGG	57,495 to 57,522	GGATATTATTACGCTCCTCTGTCGGAGG (89%; *m060R* gene)		14,900 to 14,873	GGATATTATTACGCTCCATTATCGGAGG (100%; *m060L* gene)	
						60,320 to 60,347	GGATATTATTACGCTCCTCTGTCGGAGG (25/28, 89%; *m060R* gene)	
	Probe (HEX-BHQ1)	CTGATAAGTACCCCTTATCTACAAAAACGGGTG	57,639 to 57,607	CTGCCAAATATCCCTTATCCACGCAAATGGGAG (73%; *m060R* gene)		14,756 to 14,788	CTGATAAGTACCCCTTATCTACAAAAACGGGTG (100%; *m060L* gene)	
						60,471 to 60,320 *	(No significant similarity; *m060R* gene)	

* Region amplified by the primers, ^#^ No signal due to the lack of amplification due to the relative position of the primers 2,946 bp apart; underlined nucleotides indicate no homology between primer sequence and target sequence.

**Table 3:** Optimized primers and probes’ concentrations for field samples in the mPCR.

	Concentrations in the Amplification Reaction (μM)		
Gene	Primer Fw	Primer Rv	Probe
*m000.5L/R*	0.08	0.08	0.07
*m009L*	0.12	0.12	0.14
*m060L*	0.08	0.08	0.1
*18S rRNA*	0.08	0.08	0.1

**Table 4:** Optimized protocols for quadruplex PCR with Taqman probes, and triplex PCR using EvaGreen.

Amplification Protocols for mPCR	
Taqman System	Evagreen System
Step 1: 95 °C for 4:00 Step 2: 94 °C for 0:30 Step 3: 60 °C for 0:30 Step 4: 72 °C for 0:30 Go to step 2: Repeat 2× Step 5: 94 °C for 0:30 Step 6: 60 °C for 0:30 Plate read Step 7: 72 °C for 0:30 Go to step 5 Repeat 36×	Step 1: 98 °C for 2:00 Step 2: 98 °C for 0:05 Step 3: 60 °C for 0:15 Go to step 2: Repeat 39× Melt curve: 65–95 °C (0.3 °C increment, 5 s/step)

**Table 5:** Repeatability and reproducibility of the mPCR.

Target	N. of Copies/Reaction	Intra-Assay (Quadruplicates)			Inter-Assay (2 Independent Assay with Duplicates)		
		Mean	SD	CV%	Mean	SD	CV%
pM000.5L/R	9.0 × 10^7^	11.74	0.17	1.47	-	-	-
	9.0 × 10^6^	15.23	0.13	0.83	15.13	0.32	2.12
	9.0 × 10^5^	19.09	0.02	0.10	-	-	-
	9.0 × 10^4^	22.08	0.10	0.44	22.68	0.45	1.98
	9.0 × 10^3^	25.62	0.14	0.54	-	-	-
	9.0 × 10^2^	29.00	0.45	1.55	29.53	0.27	0.91
	9.0 × 10^1^	32.86	0.27	0.82	-	-	-
	9.0 × 10^0^	36.04	1.27	3.52	36.24	1.21	3.34
pM060L	9.0 × 10^7^	13.25	0.11	0.83	-	-	-
	9.0 × 10^6^	16.89	0.17	0.99	16.72	0.25	1.50
	9.0 × 10^5^	20.55	0.14	0.69	-	-	-
	9.0 × 10^4^	23.09	0.12	0.53	23.32	0.27	1.16
	9.0 × 10^3^	27.04	0.08	0.31	-	-	-
	9.0 × 10^2^	30.05	0.06	0.20	30.82	0.32	1.04
	9.0 × 10^1^	34.52	0.42	1.21	-	-	-
	9.0 × 10^0^	37.91	1.29	3.40	37.52	1.32	3.52
pM009L	9.0 × 10^7^	11.32	0.41	3.65	-	-	-
	9.0 × 10^6^	14.15	0.35	2.47	14.74	0.36	2.44
	9.0 × 10^5^	18.10	0.07	0.40	-	-	-
	9.0 × 10^4^	21.21	0.03	0.16	21.53	0.38	1.76
	9.0 × 10^3^	24.32	0.10	0.40	-	-	-
	9.0 × 10^2^	28.21	0.17	0.59	29.14	0.43	1.48
	9.0 × 10^1^	32.47	0.63	1.92	-	-	-
	9.0 × 10^0^	35.91	0.92	2.56	35.73	1.13	3.16

**Table 6:** Comparison of the melting temperatures obtained for the singleplex and multiplexes.

	In Uniplex Format			In Triplex Format		
PCR system	*m000.5L/R*	*m060L*	*m009L*	*m000.5L/R*	*m060L*	*m009L*
Mean ± SD	80.07 ± 0.08	75.58 ± 0.10	82.54 ± 0.28	80.11 ± 0.24	75.43 ± 0.18	82.34 ± 0.31

**Table 7:** Comparison of the qualitative results obtained with the *m000.5L/R* singleplex and quadruplex.

	Singleplex (*m000.5L/R*)	Quadruplex (*m000.5 L/R*, 18S rDNA, *m060L* or *m009L*)
True positive	41 (24 ha-MYXV positive and 17 classic MYXV positive)	40 *
True negative	19	19
False positive	0	0
False negative	0	1 *

* One sample in the quadruplex analysis was positive for *m000.5L/R* (Cq 37.2) and *18S rRNA*, negative to *m009L* and *m060L*. Results from the singleplex: positive for *m009L*, negative for *m060L*).

## Data Availability

Data are contained within the article and in the Supplementary Table S1.

## Supplementary Materials

The following are available online at https://www.mdpi.com/article/10.3390/ijms222112052/s1, Table S1: In silico and in vitro analyses of the qPCR systems against representative sequences retrieved from NCBI database.
