# Supplementary material for: A Quadruplex qPCR for Detection and Differentiation of Classic and Natural Recombinant Myxoma Virus Strains of Leporids
Source: Int J Mol Sci. 2021 Nov 7;22(21):12052. doi: 10.3390/ijms222112052 (PMC8584577; doi:10.3390/ijms222112052)
Supplement: Supplementary file 1 [file ijms-22-12052-s001.zip › ijms-1380620-supplementary.pdf]

Table S1. *In silico* and *in vitro* analyses of the qPCR systems against representative sequences retrieved from NCBI database.

Strain / Isolate	Country	Year	Ac. Number	AMPLIFICATION WITH *m000.5L/R* system	*m009L* system	*m060L* system	Type of analysis
Brazil/Campinas/1949/1	Brazil	1949	KY548791	Yes	Yes	No	*In silico*
Brazil/Campinas/1949/1	Brazil	1949	JX565570	Yes	Yes	No	*In silico*
Lausanne	Switzerland	1949	KY548791	Yes	Yes	No	*In silico*
SLS/1950 (Moses strain/strain B)	Brazil	1950	JX565574	Yes	Yes	No	*In silico*
SLS (Moses strain/strain B)	Brazil	1950	JX565574	Yes	Yes	No	*In silico*
MSW, California/San Francisco 1950	California	1950	KF148065	No	No	No	*In silico*
Aust/Dubbo/2-51/1	Australia	1951	JX565567	Yes	Yes	No	*In silico*
Aust/Dubbo/2-51/1	Australia	1951	JX565567	Yes	Yes	No	*In silico*
Aust/Corowa/12-52/2	Australia	1952	JX565569	Yes	Yes	No	*In silico*
Aust/Corowa/12-52/2	Australia	1952	JX565569	Yes	Yes	No	*In silico*
Aust/Uriarra/2-53/1	Australia	1953	JX565577	Yes	Yes	No	*In silico*
Aust/Uriarra/2-53/1	Australia	1953	JX565577	Yes	Yes	No	*In silico*
England/Cornwall/4-54/1	Cornwall, UK	1954	JX565566	Yes	Yes	No	
England/Sussex/9-54/1	Sussex, UK	1954	KC660084	Yes	Yes	No	*In silico*
England/Sussex/9-54/1	UK	1954	KC660084	Yes	Yes	No	*In silico*
England/Cornwall/4-54/1	UK	1954	JX565566	Yes	Yes	No	*In silico*
England/Nottingham/4-55/1	Nottingham, UK	1955	JX565572	Yes	Yes	No	*In silico*
Belfast/1955	Ireland	1955	KY548792	Yes	Yes	No	*In silico*
England/Nottingham/4-55/1	UK	1955	JX565572	Yes	Yes	No	*In silico*
Belfast	Ireland	1955	KY548792	Yes	Yes	No	*In silico*
Germany/Munich/1985	Germany	1985	KP723387	Yes	No	No	*In silico*
Poland/ZA/1985	Poland	1985	KP723386	Yes (1 mis A/G in the probe)	Yes	No	*In silico*
Aust/NSW/Avenel/11-1990	Australia	1990	MK388104	Yes	Yes	No	*In silico*
Aust/Meby/8-91	Australia	1991	JX565571	Yes	Yes	No	*In silico*
Aust/Gungahlin/1-91	Australia	1991	JX565568	Yes	Yes	No	*In silico*
Aust/Southwell Hill/9-92/1	Australia	1992	JX565576	Yes	Yes	No	*In silico*
Aust/Bendigo/7-92	Australia	1992	JX565565	Yes	Yes	No	*In silico*
Aust/Southwell Hill/9-92/1	Australia	1992	JX565576	Yes	Yes	No	*In silico*

Aust/Bendigo/7-92	Australia	1992	JX565565	Yes	Yes	No	*In silico*
Aust/Brooklands/4-93	Australia	1993	JX565562	Yes	Yes	No	*In silico*
Aust/Brooklands/2-93	Australia	1993	JX565563	Yes	Yes	No	*In silico*
Aust/Southwell Hill/2-93	Australia	1993	JX565575	Yes	Yes	No	*In silico*
Aust/Southwell Hill/11-93	Australia	1993	KC660085	Yes	Yes	No	*In silico*
Aust/Brooklands/4-93	Australia	1993	JX565562	Yes	Yes	No	*In silico*
Aust/OB1/Hall/3-94	Australia	1994	JX565573	Yes	Yes	No	*In silico*
Aust/OB3/Hall/2-94	Australia	1994	KC660083	Yes	Yes	No	*In silico*
Australia/Woodstock 1/3-94	Australia	1994	JX565578	Yes (1 mis A/G in the probe)	Yes	No	*In silico*
Aust/Woodstock 1/3-94	Australia	1994	JX565579	Yes	Yes	No	*In silico*
Aust/Brooklands/1-95	Australia	1995	JX565564	Yes (1 mis A/G in the probe)	Yes	No	*In silico*
Aust/OB2/Hall/11-95	Australia	1995	KC660081	Yes	No	No	*In silico*
Aust/Woodstock 6/11-95	Australia	1995	JX565580	Yes	Yes	No	*In silico*
Aust/OB2/Hall/11-95	Australia	1995	KC660081	Yes	No	No	*In silico*
Myxoma virus strain 6918	Spain	1995	EU552530	Yes	No (1 mis C/A in the rev primer, 3’ end)	No	*In silico*
Aust/OB3/Hall/2-96	Australia	1996	KC660082	Yes	Yes	No	*In silico*
Aust/Southwell Hill/2-96	Australia	1996	JX565583	Yes	No	No	*In silico*
Aust/Bulloo Downs/11-99	Australia	1999	JX565584	Yes	No	No	*In silico*
Aust/Bulloo Downs/12-99	Australia	1999	KC660079	Yes	No	No	*In silico*
Aust/Bulloo Downs/12-99	Australia	1999	KC660079	Yes	No	No	*In silico*
Aust/Bulloo Downs/11-99	Australia	1999	JX565584	Yes	No	No	*In silico*
Germany/2604/2004	Germany	2004	KP723389	Yes	Yes	No	*In silico*
Germany/FLI-H/2004	Germany	2004	KP723390	Yes	Yes	No	*In silico*
Germany/3207/2007	Germany	2007	KP723388	Yes	Yes	No	*In silico*
Scotland/Perthshire/1526/2008	Scotland	2008	KY548795	Yes	Yes	No	*In silico*
England/Yorkshire/127/2008	England	2008	KY548811	Yes	Yes	No	*In silico*
Aust/ACT/Acton/07-2008	Australia	2008	MK388112	Yes	No	No	*In silico*
Yorkshire/127	UK	2008	KY548811	Yes	Yes	No	
Scotland/ Perthshire/1754/2009	Scotland	2009	KY548797	Yes	Yes	No	*In silico*

England/Yorkshire/135/2009	England	2009	KY548812	Yes	No (insertion of a C, reverse primer)	No	*In silico*
Perthshire/1754	Scotland	2009	KY548797	Yes	Yes	No	*In silico*
Scotland/ Perthshire/2080/2010	Scotland	2010	KY548802	Yes	Yes	No	*In silico*
Scotland/ Perthshire/2256/2011	Scotland	2011	KY548806	Yes	Yes	No	*In silico*
England/Yorkshire/Col/2011	England	2011	KY548813	Yes	No (Insertion of a C, reverse primer)	No	*In silico*
Scotland/ Perthshire/2409/2012	Scotland	2012	KY548807	Yes	Yes	No	*In silico*
Aust/ACT/Acton/12-2012	Australia	2012	MK388108	Yes	No	No	*In silico*
Aust/NSW/Euchareena (19)/10-2012	Australia	2012	MK388101	Yes	No	No	*In silico*
Aust/SA/Turretfield(2992)/06-2012	Australia	2012	MK388103	Yes	No	No	*In silico*
Scotland/ Perthshire/2524/2013	Scotland	2013	KY548810	Yes	Yes	No	*In silico*
Aust/ACT/Mulligans Flat/04-2013/2	Australia	2013	MK388093	Yes	No	No	*In silico*
Aust/NSW/Euchareena (3)/02-2013	Australia	2013	MK388096	Yes	No	No	*In silico*
Aust/SA/Monarto Zoo/11-2013	Australia	2013	MK388114	Yes	Yes	No	*In silico*
Aust/SA/Wilpena/11-2014	Australia	2014	MK388124	Yes	No	No	*In silico*
Aust/NSW/Carwoola/12-2014	Australia	2014	MK388117	Yes	No	No	*In silico*
Aust/NSW/Murrumbateman/10-2014	Australia	2014	MK388121	Yes	No	No	*In silico*
Aust/SA/Waterfall Gully/09-2015	Australia	2015	MK388138	Yes	No	No	*In silico*
Aust/SA/Olympic Dam/08-2015	Australia	2015	MK388142	Yes	No	No	*In silico*
Aust/SA/Qualco/01-2015	Australia	2015	MK388120	Yes	No	No	*In silico*
Aust/NSW/Deniliquin/02-2015	Australia	2015	MK388118	Yes	No	No	*In silico*
Aust/SA/Mt Gambier/09-2015	Australia	2015	MK388135	Yes	No	No	*In silico*
Aust/SA/Kangarilla/09-2015	Australia	2015	MK388136	Yes	Yes	No	*In silico*
Aust/ACT/Symonston/01-2016	Australia	2016	MK388125	Yes	No	No	*In silico*
ha-MYXV / MYXV-Tol08/18	Spain	2018	MK836424 MK340973	Yes	No	Yes	*In silico* and *in vitro*
Myxo-RHD Plus	-	-	-	Yes	No	No	*In vitro*
Dervaximyxo SG 33	France	2009	GQ409969	Yes	Yes	No	*In silico*
Mixohipra-H VMI30	-	-	-	Yes	No	No	*In vitro*

POX-LAP, strain Leon-162	-	-	-	Yes	No	No	*In vitro*
Dercunimix SG33	-	2009	GQ409969	Yes	Yes	No	*In silico*
Rabbit fibroma virus (Kasza strain of SFV)		1974	NC_001266	No	No	No	*In silico*
Mixohipra FSA	-	-	-	No	No	No	*In vitro*
Mixovacina	-	-	-	No	No	No	*In vitro*
Lyomyxovax	-	-	-	No	No	No	*In vitro*

*In silico result meaning: Yes- primers and probe target sequences are 100% conserved. No- lack of amplification in the *in silico* PCR at 55ºC FASTPCR 6.7 (PrimerDigital, 2020). mis-mismatch, D- doubtful.
